# Dr. Chas. Bonsall’s Address before the Mississippi Valley Association of Dental Surgeons

**Published:** 1854-04

**Authors:** Chas. Bonsall


Dr. CHAS. BONSALL'S *Address before the Missisippi Valley Association of Dental Surgeons.*

GENTLEMEN AND FELLOW MEMBERS—It gives me pleasure to meet once more so many of my professional brethren, drawn together by a general intercst in the advance and improvement of our commen profession.

It is now *ten* years since a few of *us*, together with some others, first met in this city in convention, for the purpose of forming an association for the purpose of mutual improvement, and the promotion of dental science, and I flatter myself that there is no one present—at least no one who was in practice at that time—but who will acknowledge that the consequences of associating together have been beneficial to us all, both as to improvement in the science and practice of our profession, and also perhaps still more in in promoting the exercise of that gentlemanly courtesy, which should characterize members of a liberal profession, in both *social* and *professional* intercourse.

Probably most of the older members of the Association can recollect how uncommon it was ten or twelve years ago, to find any real friendly feeling or willingness to communicate knowledge from one dentist to another ; on the contrary, the moment any one practitioner entered the office of another, he met with a *cool*, though sometimes *civil* welcome (it so it could be called); and with evident care to prevent him from seeing or learning any of the secrets of the office, he was bowed out, as soon as he could be decently got shut off.

Now, on the other hand, at least so far as my observation has enabled me to judge, there seems to be an almost universal willingness, among those who are at all worthy of the name of Dentist, to impart whatever information they may be able to give to their fellow practitioners ; and also a perceptible improvement in the manner of speaking of their neighbor dentist, when it can be done with propriety, as well as the cordial manner in which members of the profession meet each other, comparing so favorably with what was customary fifteen or twenty years ago.

It was very pleasant to me, last summer, at the annual meeting of the American Society, where I was personally acquainted with but few of those present, to find so much cordiality among those in attendance, even where they had met for the first time ; and after the adjournment, as I occasionally, while, in the Eastern cities, found time to call for a few minutes on different dentists whom I knew to stand well professionally, I almost invariably met with a pleasant welcome, and apparent anxiety to impart information.

It should be a momentous question with each of us, how shall we act, so as to do our part, to elevate the character of the profession—and what means can we use to raise its reputation to the hight which it deserves to hold, taking into view its importance to the human family.

I am entirely satisfied that one of the prominent means, within the reach of all, is to determine to be strictly honest in all their operations, and never determine to perform an operation for the patient applying, unless they feel comparatively certain that it will be beneficial to him or her and creditable to themselves.

I also think we can, each one among his own patients, while advising with them, give such explanations and reasons as will be understood by them, to show that their teeth are more valuable to them than they had heretofore supposed—and that it is really a matter of great importance to them to take great care of these organs, and when requiring attention to call in the advice and aid of the best dental talent within their reach.

If we would follow always the best course within our knowledge, without allowing ourselves to be dismayed by difficulties requiring time to overcome, or the fear of giving pain, which, though an *amiable* trait, may be carried too far, or, on the other had, a fear to charge what will be a sufficient recompense for the time and material employed, we should, in a few years, find that our patients had begun to place their dentist in the rank he deserves to fill, in relation to the practitioners of other branches of the healing art.

If any of us should have a call from a patient, who can not afford to pay a sufficient price to compensate us for the time and material necessary to a good operation, it would be far better for our own, and the reputation of the profession to decline operating, unless we feel willing to make the necessary sacrifice, and take the balance of our pay in the satisfaction of having done well for the patient. In such cases I would advise that the full charge should be mentioned, and let it be understood that a part of the bill is gratuitous.

Among other great advantages which we enjoy over what we possessed a few years ago, is the large amount of dental periodical literature now at our command, diffusing most of the improvements in dental science or practice through the profession over the country, in a comparatively short time—and while alluding to this subject, let me give my opinion that we *are*, or *ought to be*, all students, and that none of us have made such progress that we have nothing new to learn; on the contrary, I believe we all will find hints sufficient in each year of the Dental Register, Journal, News Letter, Recorder, etc., to fully pay us for the cost of subscription.

One of the most pleasing and improving features of our dental associations, to those who attend the annual gatherings, is, in my opinion, the interlocutory meetings, in which each member gives his own experience as to any peculiar mode of practice, which may be under diccussion; and at these meetings, I am satisfied that the older membrs of the profession are benefitted perhaps almost equally with the younger.

While speaking of advantages to be gained by members of our Dental Associations, I would wish to impress upon us all the necessity of being very particular to satisfy ourselves fully as to the qualifications and character of each one, who may be proposed as a member at any time, as it is only by maintaining a high standard that we can benefit ourselves and the profession, or, in the words of the lamented Hayden, at the first meeting of the American Society, some fifteen years ago —"Gentlemen, if you would make your society respectable, and would have it esteemed so by the world, do not be hasty in electing your members, but be careful and judicious in selecting those whom you would admit into your ranks."

This advice will be found equally good now.

The public—at least a portion of them, I believe—are beginning to know the difference between the well-educated, conscientious dentist, and those who, really knowing nothing of the anatomy or physiology of the parts implicated, hope to gain practice by their flaming advertisements, and strong promises of the wonderful operations they can perform.

I would urge upon every dentist who has the respectability of his profession at heart, to be very careful as to the character and capability of any applicant as a student, before receiving him, and then to require, at least, two year's pupilage in the office; so as to have some prospect of his being tolerably well qualified to operate before he is turned out on the world as being competent to operate successfully.

I suppose most of our older dentists will agree with me in their experience, that even, after five or six years of close practice, there is still much to be learned; if this is correct, then how foolish to suppose that a few weeks or months in the office of a dentist is sufficient to learn, how to practice successfully. So that if we wish to have our profession respected, we must all use our endeavors to break up the system of taking students for short periods, no matter how large an office-fee they may be willing to offer.

I myself have had frequent applications from young gentlemen, some of whom tell me that they have been practicing for some years, but wish a little further instructions. I invariably tell them that I should require them to remain at least *two years* in the office—and longer, unless I should think them competent at that time; and this usually closes the conference very soon, as they generally wish to get to practising in a few weeks or months.

Let us all leave selfish considerations out of view, and look only to the honor of our profession, and the benefit we can be of to our fellows, and ere long, if not at present, we shal receive the credit which will be due to us.

Gentlemen — I had intended to have written much more, and on other subjects connected with the interests of our Association; but any effort of this kind is so entirely unpleasant to me, that I had put it off till the last moment, and now within the last two days, other engagements, which were unavoidable, have prevented my being able to carry out the intention.

I have no doubt, however, that you will feel satisfied that there is quite enough, unless it were better worth hearing—so that with merely saying that it is because of my great anxiety for the honor and benefit of the profession, that I have ventured so much in the way of advice, I will close, wishing that we all, when about to retire from the dental profession, or upon the close of life, may have the conviction that we have done our duty to our patients and the profession.

Chas. Bonsall.

